# Do conditioning focused various-sided training games prepare elite youth male soccer players for the demands of competition?

**DOI:** 10.5114/biolsport.2022.109454

**Published:** 2021-10-25

**Authors:** Michael G. Sydney, Martin Wollin, Dale W. Chapman, Nick Ball, Jocelyn K. Mara

**Affiliations:** 1 University of Canberra Research Institute for Sport and Exercise (UCRISE), Canberra, Australia; 2 School of Science, Faculty of Science & Technology, University of Canberra, Australia; 3 School of Health Sciences, La Trobe University, Melbourne, Australia; 4 New South Wales Institute of Sport (NSWIS), Sydney, Australia; 5 University of Canberra Research Institute for Sport and Exercise (UCRISE), Canberra, Australia; 6 Discipline of Sport and Exercise Science, Faculty of Health, University of Canberra, Australia

**Keywords:** Performance, Coaching, Game Analysis, Team Sport, Youth

## Abstract

Relative metrics (i.e. distance covered per minute of match time) are regularly used to quantify soccer player movement demands. However, limited literature is available concerning the peak player demands during training. This study aimed to compare the relative and peak demands of conditioning-focused various-sided training games (VSG) to competition matches in elite youth male soccer players according to playing position. Data from twenty-nine competition matches (national) and twenty-two VSGs (small, medium, and large) were collected for twenty-three elite under-17 soccer players using 15-Hz portable global positioning system tracking devices (GPSports, Canberra, Australia). Relative player movements were reported as total distance (TD) and high-speed running distance (HSRD) (> 5.0 m/s) per minute of total playing time. Peak player movements were calculated using a 1-minute rolling epoch length, reported as the maximum TD and HSRD. Linear mixed models demonstrated interactions between VSG type and player position for relative TD (*p* < 0.001) and HSRD (*p* < 0.001), and peak TD (*p* = 0.010) and HSRD (*p* = 0.003). The relative TD of VSGs were greater than match-play for all player positions. However, only Central Defenders demonstrated similar HSRD in MSGs and LSGs compared to match-play when analysed using relative calculations. External Attackers also replicated match-play relative HSRD demands in LSGs. No VSG type was found to replicate or supersede the peak player movements of match-play across any playing position. Consequently, VSGs should be supplemented with high-speed running training to prepare players for the peak running requirements of match-play.

## INTRODUCTION

Soccer is a team sport characterised by high technical and tactical demands as well as large external workloads pertaining to total distance (TD) covered and player movements at high-speed (> 5.0 m/s) [1–4]. To optimise player readiness for the rigors of match-play, knowledge of movement demands concerning positional groups is vital for coaches and practitioners to use as evidence-based parameters for training programme design and player monitoring [5–7]. Considerable research has investigated the difference between the physical demands of various-sided games (VSGs) and running drills as a training modality to improve player performance [8–12]. This research has reported that whilst VSGs are effective training modalities for technical and tactical development, running drills should be implemented when seeking to expose players to high-intensity running stimuli.

Previous research in elite youth male soccer players has primarily focused on quantifying the absolute and relative (i.e. distance covered per minute of match time) movement demands during match-play and training games with few studies reporting peak running demands [3, 7, 11–13]. Dalen et al. [11] assessed the difference in the mean and peak total distance, high-intensity running (> 5.5 m/s) and sprinting (> 7.0 m/s) during 4v4 and 6v6 (plus goal keepers) VSGs to the demands of match-play in elite senior (mean age = 24.9 ± 4.2) Norwegian soccer players utilising 5-minute rolling epochs. It was reported that high-intensity running distance during 4v4 and 6v6 was 78% and 86% lower than in the peak period of match-play and 50% and 67% lower than mean match-play values, indicating that 4v4 and 6v6 VSGs could not be implemented to elicit high-intensity running stimuli [11]. However, comparison between mean and peak running demands in large-sided games (i.e. 7v7, 8v8 and 9v9) and match-play remain unclear. Furthermore, as this study utilised elite senior male soccer players, the results may not be transferable to an elite youth cohort. As such, data elucidating the peak movement demands of match-play and VSGs including large player numbers may assist coaches and practitioners adjust training sessions according to player availability, optimise player readiness and provide valid training markers for conditioning training stimuli in elite youth male soccer players [5–7].

A common training strategy for soccer coaches and practitioners is to prescribe VSGs as a holistic training modality to effectively address physical, technical and tactical training objectives simultaneously [7, 9, 10, 12]. However, if the objective of conditioning training is to replicate or overload the movement demands exhibited during match-play, use of a holistic training concept that equally prioritises technical and tactical objectives could result in an inadequate stimulus to achieve conditioning objectives [7, 9, 10, 12]. Furthermore, to ensure maximal transfer to match-day performance, the principle of specificity suggests that VSGs used in soccer conditioning training should reflect the relative field dimensions (length to width ratios) of regulation playing fields [7, 9, 10, 12]. However, previous studies have used arbitrary field dimensions, total pitch areas (m^2^) and relative pitch area per player sizes (m^2^) and therefore the relationship between peak demand running metrics in VSGs utilising relative field dimensions requires further investigation [7, 9, 10, 12]. Novel data pertaining to peak player movements during conditioning-focused various-sided training games (VSGs) and match-play can help develop age-appropriate training metrics and determine whether such training drills are valid player conditioning stimuli or if supplementary conditioning drills are required [7, 9, 10, 12]. Thus, the aim of this study was to identify and differentiate the relative and peak player movement demands of conditioning-focused various-sided training games (VSGs) to official competition matches. This study also sought to identify the influence of player position on relative and peak movement demands. This detailed investigation is pertinent to coaches and practitioners as it may provide insight into the appropriate design of VSGs when seeking to prescribe an overload stimulus, further aiding in the optimisation of player readiness from a positional profile perspective.

## MATERIALS AND METHODS

### Experimental approach to the problem

This study employed a longitudinal observational design using a single player cohort across a thirteen-month period in national (National Youth League) and state (National Premier League) competitions. To achieve the study aims, the relative and peak running demands of players during competition matches and VSGs were compared. National Premier League (NPL) competition matches were played throughout an eight-month competition calendar period (March – October). National Youth League (NYL) competition matches were played across a three-month competition calendar (November – January). Data were only included from each competition match where the player competed in at least fifty-minutes. VSG files were only included in analysis if the player completed every VSG for that training session. The format of VSGs implemented in this study were designed by coaching staff to reflect the relative field dimensions (length to width ratios) of regulation playing fields. During the season players typically participated in 4–5 soccer specific on-field sessions, 1–2 strength and conditioning sessions and 1–2 competition matches per week. Players also participated in 1–2 post-match contrast water immersion recovery sessions per week either following a training session or competition match. Conditioning-focused VSGs were scheduled three days prior to competition matches once a week as per microcycle training plans designed by coaching and practitioner staff. The type of VSG employed during each microcycle was at the discretion of coaching staff. Environmental conditions, competition match and training times varied substantially throughout the data collection period in accordance with seasonal fixtures and phase of season (i.e. pre-season, competition etc.). Players were familiarised with all VSG formats and task constraints as a part of regular training instructions from coaching staff.

### Participants

Twenty-three elite youth male soccer players (age: 15.6 ± 0.8 years, height: 173.4 ± 5.1 cm; body mass: 65.2 ± 6.0 kg, Yo-Yo Intermittent Running Test Level 2 distance: 809 ± 248 m) from the Australian under-17 National Centre of Excellence program participated in this study. Participants were classified elite, as they were selected to the National Centre of Excellence program and participated in the National Youth League (NYL) competition, the highest standard of domestic competition for their age group and represented their country at international age level. All participants and their parents or legal guardians were informed about the study protocol, requirements, benefits, and risks before giving their written informed consent to participate. All procedures were conducted in accordance with the Declaration of Helsinki and approved by the University of Canberra Human Research Ethics Committee.

### Procedures

Competition matches (n = 29) observed in this study were National Youth League (n = 8) and National Premier League (n = 21) fixtures. The National Youth League is a youth professional development competition whilst the National Premier League is an adult (senior) semi-professional competition. A 4**-**3**-**3 formation was used in all matches throughout the data collection period. Matches were played on 100 × 60 m field dimensions on natural (n = 28) and synthetic (n = 1) turf surfaces. Each match was ninety-minutes in duration, separated into two forty-five-minute halves, with any additional time determined by the match referee. All matches were played under the same competition rules, limiting each team to three substitutions and a fifteen**-**minute break for half time. Matches were preceded by a thirty-minute standardised warm up consisting of small-sided games (SSGs), short and intermediate length maximal sprint efforts, short and long passing, shooting, as well as dynamic stretching.

Participants were categorised according to playing position as directed by the head coach. Playing positions were CD = Central Defenders (n = 4), ED = External Defenders (n = 5), MD = Mid-fielders (n = 6), EA = External Attackers (n = 5) and CA = Central Attackers (n = 3). Some players featured in more than one playing position across competition fixtures with their position being defined for each individual competition match accordingly. Each player competed in an average of twelve competition matches (range = 3–27).

Conditioning-focused VSGs were divided into three categories: Small-sided games (SSG), medium-sided games (MSG) and large-sided games (LSG). The design details of each game type are provided in Table 1. The relative pitch area per player (m^2^) for each VSG type was calculated as the total pitch area divided by the number of players. The length to width ratio of each VSG type was 5:3. This was calculated based on the length to width ratio of regulation soccer field dimensions to ensure the environment of VSGs was synonymous to match-play. Goalkeepers were present for each game type although they were excluded in the calculations when determining the relative pitch area per player (m^2^) and length to width ratio. Like competition matches, VSGs were preceded by a thirty-minute standardised warm up, consisting of short and intermediate length maximal sprint efforts, short and long passing, shooting, as well as dynamic stretching.

**TABLE 1:** Various-Sided Games and Competition Match Design

No. of Players*	Training Prescription	Field Dimensions [Length × Width (m)]	Total Pitch Area (m^2^)	Relative Pitch area per player (m^2^)
**Small-Sided Games**				
3v3*	12 × 2-min	30 × 18	540	90
5v5*	12 × 2.5-min	50 × 30	1500	150

**Medium-Sided Games**				
6v6*	10 × 5-min	60 × 36	2160	180
7v7*	4 × 10-min	70 × 42	2940	210

**Large-Sided Games**				
8v8*	2 × 10-min	80 × 48	3840	240
9v9*	2 × 10-min	90 × 54	4860	270

**Competition Matches**				
10v10*	2 × 45-min Halves	100 × 60	6000	340

* Excludes Goalkeepers.

VSGs were organised and administered by the coaching staff during each training session. Participating players were purposefully chosen to ensure a balance of playing positions amongst selected teams. The primary training objective of the VSGs was player physical conditioning, with technical and tactical development being secondary objectives. Each game comprised of two full-sized goals with the addition of a goalkeeper for each team. Spare balls were kept in the goal of both teams. The goalkeeper was responsible for a fast restart of play if the ball exited the field of play, or a goal was scored. Players started the games in appropriate playing formations for each VSG type as determined by the coaching staff, with the teams alternating who started with possession of the ball. VSGs used the same playing rules as competition matches except for the offside rule, corner kick and the kick-off to restart the game. Coach feedback was present during each VSG and players were instructed to pressure the opposition as much as possible. The number of observations of each game type according to player position are provided in Table 2.

**TABLE 2:** Number of Observations of Each Game Type According to Player Position.

Player Position	SSG		MSG		LSG		Competition Matches

	3v3	5v5	6v6	7v7	8v8	9v9	10v10
Central Defender	78	32	45	19	70	6	56
External Defender	69	39	20	31	100	11	56
Midfielder	137	46	59	32	118	11	82
External Attacker	136	31	70	32	120	8	54
Central Attacker	43	7	15	9	32	4	28
Total	463	155	209	123	440	40	276
VSG Type Total	618		332		480		

### Data collection

The movement demands of players during VSGs and competition matches were captured using commercial 15-Hz portable global positioning system (GPS) tracking devices (SPI HPU, GPSports, Canberra, Australia). Players were fitted with a garment that allowed the GPS unit to be positioned between the scapulae. Each player was allocated the same GPS unit for the duration of data collection to minimise the effect of inter-unit error. After each competition match and VSG was completed, GPS data were extracted using proprietary software (Team AMS, Canberra, Australia). Each GPS file was processed to include only data captured during the VSG and competition match time (i.e. warm-up data were excluded from the analysis). To ensure satellite connectivity, GPS devices were turned on thirty-minutes before each VSG and competition match. During all competition matches and VSGs, 4–12 satellites were available for connectivity and signal transmission, satisfying the criteria for ideal positional detection [14]. Horizontal dilution of precision (HDOP) was not reported by the proprietary software (Team AMS, Canberra, Australia).

Movement demands were reported as total distance per minute (TD/min) and high-speed (> 5.0 m/s) running distance per minute (HSRD/min). The HSRD velocity threshold was chosen based on recommendations for elite youth male soccer players [3, 4]. The interunit reliability, expressed as a coefficient of variation, for the GPS devices has been reported as 1.4% for total distance, 7.8% for distance at speeds between 2.0 m/s to 5.9 m/s and 4.8% for distance covered at speeds > 5.9 m/s [15]. Relative movement demands were calculated by dividing the absolute values of TD and HSRD by the duration of the competition match or VSG. To calculate peak TD and HSRD, each competition match and VSG file was split into 30 second time intervals. The *rollapply* function from the *zoo* [16] package in R version 4.0.3 [17] using RStudio version 1.4.1103 [18] was applied to calculate 1-minute rolling sums for TD and HSRD. Peak demands were defined as the maximum TD and HSRD achieved in 1-minute. Rolling epochs were employed in this investigation as fixed epochs have been demonstrated to underestimated total (7–10%) and high-speed (12–25%) distance (defined as > 5.5 m/s) in elite senior male soccer players [6]. Furthermore, whilst the use of 1-, 2-, 5- and 10-minute rolling epochs has been utilised when determining peak running demands [6, 19], the duration of 3v3 (2-min) and 5v5 (2.5-min) SSGs as well as 6v6 (5-min) MSGs in this study do not allow for 5- and 10-minute rolling epochs to be applied. As such, to allow comparison and account for the differing durations between VSGs and competition matches, 1-minute rolling epochs were employed [6, 19].

### Statistical analysis

Statistical analyses were conducted using R version 4.0.3 [17] in RStudio version 1.4.1103 [18]. Using the *lmer* function from the *lme4* package [20], separate Linear Mixed Models (LMM) were applied to determine the difference in the relative and peak movement demands (TD, HSRD) (dependent variables) between game types and player positions (fixed factors). Repeated measures for each player in different game types, as well as the repeated measures introduced by multiple sets of the same VSG during training sessions were treated as random factors. A Type II Wald F test was conducted using the *Anova* function from the *car* package [21] to determine the significance of any interaction and main effects between game types and player position (alpha level = 0.05). The assumptions of homoscedasticity and linearity were determined upon visual inspection of plots of the fitted values against the residuals [22]. To account for multiple comparisons between substitution status, playing positions and epoch lengths, p-values were adjusted with the Benjamin-Hochberg adjustment, applied using the *p.adjust* function [23, 24]. The assumption of normality was determined upon visual inspection of histograms and Q-Q plots of the residuals [25]. Relative and peak HSRD were transformed using a natural logarithm as they violated the assumption of normality of residuals. These log-transformed variables were then used as the dependent variables in their respective LMM. Effects size statistics to compare the movement demands of VSG types to competition matches (according to player position) were calculated by Cohen’s *d,* using the least squares means and the pooled standard deviation of the random effects to account for the structure of the LMM [26]. For relative and peak HSRD the log-transformed variables were used in the effect size calculations. The effect sizes were interpreted as trivial: |*d*| < 0.2, small: |*d*| 0.2–0.49, moderate: |*d*| 0.5–0.79 and large: |*d*| ≥ 0.8 [27].

## RESULTS

**FIG. 1:**
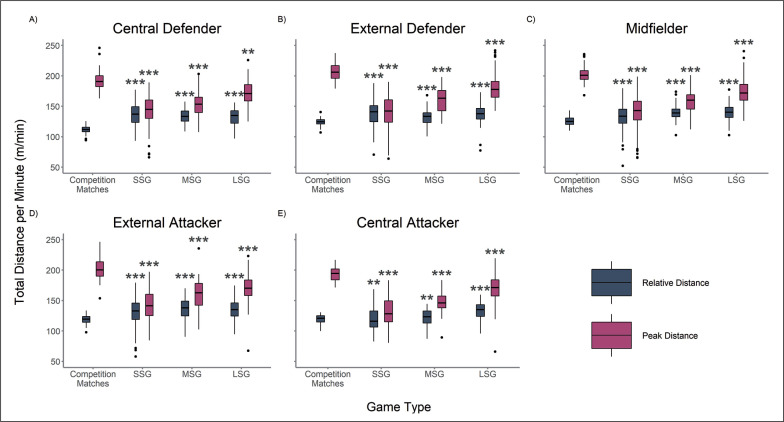
Relative and Peak Total Distance (TD) for Central Defenders (A), External Defenders (B), Midfielders (C), External Attackers (D) and Central Attackers (E) According to Game Type. * |*d*| 0.2–0.49 small effect when compared to match-play. ** |*d*| 0.5–0.79 medium effect when compared to match-play. *** |*d*| ≥ 0.8 large effect when compared to match-play.

**FIG. 2:**
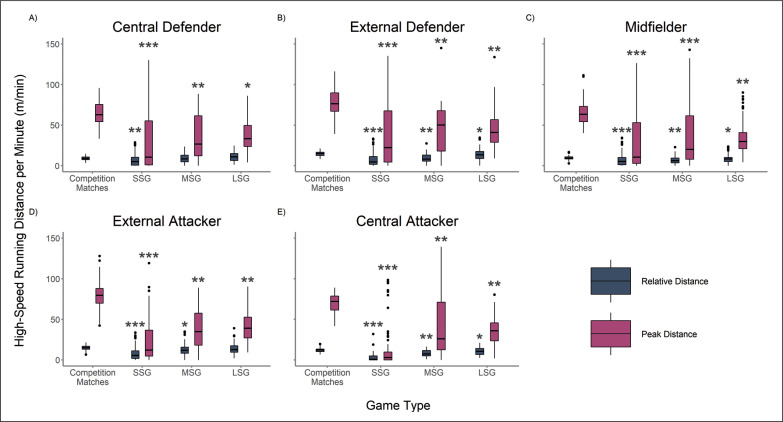
Relative and Peak High-Speed Running Distance (HSRD) for Central Defenders (A), External Defenders (B), Midfielders (C), External Attackers (D) and Central Attackers (E) According to Game Type. * |*d*| 0.2–0.49 small effect when compared to match-play. ** |*d*| 0.5–0.79 medium effect when compared to match-play. *** |*d*| ≥ 0.8 large effect when compared to match-play.

The difference of relative and peak TD and HSRD between playing positions and game type are outlined in Figures 1 and 2. The LMM for relative movement demands demonstrated interactions between game type and player position for TD (*p* < 0.001) and HSRD (*p* < 0.001). Interactions between game type and player position for peak TD (*p* = 0.010) and HSRD (*p* = 0.003) were also found. When analysed using relative calculations, the TD of VSGs were greater than competition matches with medium and large effect sizes (|*d*| range = 0.50–1.74) across all player positions (Figure 1).

Furthermore, Figure 2 shows that all player positions demonstrated lower relative HSRD in SSGs and MSGs compared to competition matches with small to large effect sizes (|*d*| range = 0.28–1.92), with the exception of Central Defenders in MSGs (Figure 2 A). Central Defenders and External Attackers (Figure 2 A, D) elicited comparable relative HSRD demands in LSGs to match-play. External Defenders, Midfielders and Central Attackers covered less relative HSRD in LSGs compared to match-play with small effect sizes (|*d*| range = 0.24–0.31) (Figure 2 B, C, E). No VSG type was found to replicate or supersede the peak TD, with medium to large effect sizes (|*d*| range = 0.76–2.65), and HSRD, with small to large effect sizes (|*d*| range = 0.45–1.63), of match-play across all playing positions (Figures 1, 2).

The least-squares mean difference of relative and peak running demands and 95% confidence interval of difference according to VSG format, not accounting for playing position are presented in Tables 3 and 4, respectively.

**TABLE 3:** Pairwise Comparison of Competition Matches and Various-Sided Game Formats for Relative Physical Demands

	VSG Comparisons	Least-Squares Mean Difference of Relative Running Demands (m/min)	95% Confidence Interval of Difference (Lower-Upper)	Adjusted p Value	Percent Difference (%)	Effect Size
TD	CM vs. SSG	–18	–20 to –15	< 0.001	–16	–1.04 (Large)
	CM vs. MSG	–19	–22 to –16	< 0.001	–17	–1.14 (Large)
	CM vs. LSG	–18	–21 to –16	< 0.001	–17	–1.09 (Large)

HSRD	CM vs. SSG	5	4 to 6	< 0.001	46	1.21 (Large)
	CM vs. MSG	3	1 to 4	< 0.001	22	0.45 (Small)
	CM vs. LSG	0	–1 to 2	0.079	4	0.15 (Trivial)

CM: Competition matches, VSG: Various-sided games, SSG: Small-sided games, MSG: Medium-sided games, LSG: Large-sided games, TD: Total distance, HSRD: High-speed running distance. Percent Difference: Percent difference of the least squares means between competition matches and VSG format, relative to competition matches. Negative values denote players recording greater physical demands in VSGs whilst positive values denote players reporting greater physical demands in CMs. Cohen’s d: trivial |*d*| < 0.2, small: |*d*| 0.2–0.49, moderate: |*d*| 0.5–0.79 and large: |*d*| ≥ 0.8.

**TABLE 4:** Pairwise Comparison of Competition Matches and Various-Sided Game Formats for Peak Physical Demands

	VSG Comparisons	Least-Squares Mean Difference of Peak Running Demands (m/min)	95% Confidence Interval of Difference (Lower-Upper)	Adjusted p Value	Percent Difference (%)	Effect Size
TD	CM vs. SSG	54	50–58	< 0.001	29	2.31 (Large)
	CM vs. MSG	37	33–41	< 0.001	20	1.58 (Large)
	CM vs. LSG	24	21–28	< 0.001	13	1.05 (Large)

HSRD	CM vs. SSG	36	31–41	< 0.001	65	1.18 (Large)
	CM vs. MSG	29	24–34	< 0.001	53	0.67 (Medium)
	CM vs. LSG	33	29–37	< 0.001	60	0.53 (Medium)

CM: Competition matches, VSG: Various-sided games, SSG: Small-sided games, MSG: Medium-sided games, LSG: Large-sided games, TD: Total distance, HSRD: High-speed running distance. Percent Difference: Percent difference of the least squares means between competition matches and VSG format, relative to competition matches. Negative values denote players recording greater physical demands in VSGs whilst positive values denote players reporting greater physical demands in CMs. Cohen’s d: trivial |*d*| < 0.2, small: |*d*| 0.2–0.49, moderate: |*d*| 0.5–0.79 and large: |*d*| ≥ 0.8.

## DISCUSSION

The aim of this study was to compare the relative and peak movement demands of conditioning-focused various-sided training games (VSGs) to official competition matches. Within this analysis we also sought to identify the influence of player position on these movement demands. The results of this study indicate that, when analysed using relative metrics, SSGs, MSGs and LSGs could be used to supersede the relative TD demands of match-play (Figure 1). However, only Central Defenders and External Attackers were found to replicate the relative HSRD demands of match-play in LSGs (Figure 2 A, D). In contrast, when analysed using peak metrics, no VSG type was found to replicate or supersede the peak TD and HSRD of match-play across all playing positions (Figures 1, 2) and subsequently cannot be prescribed to prepare players for the peak running requirements of competition matches.

A player’s ability to produce high-speed running is paramount to gain an advantage in decisive attacking or defensive soccer situations and is considered a valid and important measure of physical performance in soccer [28, 29, 30]. The relative and peak HSRD observed in SSGs and MSGs were generally lower than the demands observed in matches (Figure 2). Large Sided Games elicited similar relative HSRD demands to match-play for Central Defenders and External Attackers (Figure 2 A, D) whilst External Defenders, Midfielders and Central Attackers covered slightly less relative HSRD in LSGs compared to matches. All playing positions covered less peak HSRD in SSGs, MSGs and LSGs when compared to match-play (Figure 2). The lower relative and peak HSRD in SSGs and MSGs is likely a result of decreased total and relative pitch area, reduced interpersonal distances and compact defensive behaviours, affording players less of an opportunity to perform high-speed running compared to LSGs [8, 31, 32]. The reduced peak HSRD recorded in LSGs compared to match-play could potentially be due to the time constraints, altering the conscious or subconscious pacing strategies of players or affording players less opportunity to be subjected to demanding phases of play [33].

To optimally prepare players for the demands of match-play, players must be exposed to high-speed running loads that surpass competition situations relative to the requirements of their position [11, 28, 31]. In alignment with the recommendations by Arslan et al. [10] and Köklü et al. [9] the results of this study demonstrate that supplementary high-speed running training should be prescribed and periodised appropriately in conjunction with VSGs to adequately prepare players for position-specific, match-play peak high-speed running requirements. As such, exposure to appropriate high-speed running external workloads will need to be planned for separately as selective or indicated interventions targeting either positional player subgroups or individuals who have not reached their respective required loads for physical preparation. However, this presents a scheduling challenge for coaches and practitioners, particularly if there are mid-week fixtures and off-field strength and conditioning practices to accommodate [31]. An important adjunct to this outcome is that previous research has reported that high-speed running is a modifiable risk factor for ham-string injuries [34, 35]. To allow for adequate recovery and promote optimal player readiness, exposure to an overload training stimulus should occur at least 96 hours before a competition match during microcycles [31]. By scheduling supplementary high-speed running training accordingly, coaches and practitioners may reduce risk for non-contact hamstring injuries and optimise player readiness according to the requirements of relevant playing positions [31, 35].

Whilst absolute and relative workload metrics can provide knowledge to coaches and practitioners, previous research has suggested that utilising 1-minute rolling epoch lengths to determine the peak movement demands provides a more meaningful understanding of the most demanding periods of match-play [5, 6, 11]. Moreover, when designing training drills and programmes in accordance with the principles of overload and specificity, peak movement demand metrics may provide coaches and practitioners with a more valid training marker for monitoring the intensity of their conditioning training sessions. Practically, coaches and practitioners should be aware of the differences and compare relative and peak player movement demands in VSGs and matches to help provide greater context regarding the movement demands imposed.

Whilst this study provides valuable information to coaches and practitioners, some limitations should be considered. Firstly, Novak et al. [36] recently highlighted that true peak running demands appear to be highly individual and likely occur under a multitude of conditions leading to the use of peak running metrics as training benchmarks being questioned. Therefore, coaches and practitioners should seek to examine peak running demands as a composite construct resulting from a combination of physical, technical, tactical and contextual variables [36]. Future studies should seek to quantify such variables in addition to players internal response when analysing peak running demands in VSGs to shed further light on the practicality of VSGs for conditioning training [36]. The varying duration and training prescription between VSG types is a limitation as this may have influenced the pacing strategies of players [33]. However, it could be argued that this approach seems to have greater ecological validity as the research environment reflected the training structure implemented by coaching staff. As a longitudinal study design was employed, the influence of fitness levels and fixture congestion across different periodisation phases should be considered and analysed in future investigations. Recent research has highlighted the use of analysing peak player movement demands according to ball-in-play time periods [4] but was outside of scope of this investigation. Expansion of this research to accurately identify and record the external workloads of players during ball-in-play periods during conditioning focused VSGs and match-play would be extremely beneficial to understand the minimum effective dosage needed to achieve desired overload parameters.

## CONCLUSIONS

We report on the relative and peak demands of conditioning-focused various-sided training games (VSG) in comparison to official competition matches, with consideration to the influence of player position. Players superseded the relative total running requirements of match-play in all VSG formats. However, only Central Defenders and External Attackers were able to replicate the relative high-speed requirements of match-play during LSGs. Furthermore, players were not able to replicate or supersede the peak total and high-speed running demands of match-play in any VSG format. Therefore, coaches and practitioners are not able to use VSGs to elicit an appropriate training stimulus to prepare players for the peak running requirements of match-play. Despite potential time constraints throughout microcycles and the efficiency of using VSGs as a training modality, coaches and practitioners are advised to implement supplementary high-speed running training to best prepare elite youth male soccer players for the peak running requirements of match-play. For example, the peak total and high-speed running metrics for match-play reported in this study could be used as benchmarks to develop position specific supplementary high-speed running training.
